# Carbon nanotube embedded adhesives for real-time monitoring of adhesion failure in high performance adhesively bonded joints

**DOI:** 10.1038/s41598-020-74076-y

**Published:** 2020-10-08

**Authors:** Tadej Bregar, Donglan An, Somayeh Gharavian, Marek Burda, Isidro Durazo-Cardenas, Vijay Kumar Thakur, David Ayre, Marcin Słoma, Mark Hardiman, Conor McCarthy, Hamed Yazdani Nezhad

**Affiliations:** 1 Enhanced Composites and Structures Centre, School of Aerospace, Transport and Manufacturing, Cranfield University, Cranfield, MK43 0AL UK; 2 School of Materials, The University of Manchester, Oxford Road, Manchester, M13 9PL UK; 3 Cametics Cambridge Advanced Metals Limited, Unit 24, South Cambridge Business Park, Babraham Road, Sawston, Cambridge, CB22 3JH UK; 4 Through-Life Engineering Services Institute, School of Aerospace, Transport and Manufacturing, Cranfield University, Cranfield, MK43 0AL UK; 5 Department of Engineering, Science and Technology, SRUC, Edinburgh, DG1 3NE UK; 6 Faculty of Mechatronics, Warsaw University of Technology, 00-661 Warsaw, Poland; 7 School of Engineering, CONFIRM Centre and Bernal Institute, University of Limerick, Limerick, V94 T9PX Ireland; 8 Department of Mechanical Engineering and Aeronautics, City University of London, London, EC1V 0HB UK

**Keywords:** Mechanical properties, Aerospace engineering, Mechanical engineering, Composites, Carbon nanotubes and fullerenes, Characterization and analytical techniques

## Abstract

Carbon nanotubes (CNTs) embedded polymers are of increasing interest to scientific and industrial communities for multi-functional applications. In this article, CNTs have been introduced to high-strength epoxy adhesive for enabling in-situ strain sensing in adhesively bonded aluminium-to-aluminium single-lap joints to accurately indicate the onset and propagation of adhesion failure to the evolution of piezo-resistivity in varying mechanical loads. The CNT modified adhesive in bonded joints and the CNT modified adhesive alone have been tested under monothonic and cyclic tensile loads up to ultimate failure. The changes in the piezo-resistivity induced by the CNTs have been monitored in situ with respect to loading*.* A novel interpretation method has been developed for progressive, instantaneous adhesion failure estimation under cyclic tensile stresses from a resistivity baseline. The method indicates that the in-situ resistivity changes and the rate of the changes with strain, i.e. sensitivity, strongly correlate with the adhesion failure progression, irrespective of the CNT dispersion quality. Moreover, the effect of bond thickness on the evolution of piezo-resistivity and adhesion failure have been studied. It was observed that relatively thin adhesive bonds (0.18 mm thickness), possessing higher CNT contact points than thick bonds (0.43 mm thickness), provide 100 times higher sensitivity to varying cyclic loads.

## Introduction

Carbon nanomaterials have been receiving vast interest in multifunctional applications, among which carbon nanotubes (CNTs) are one of the most researched ones. Their exceptional mechanical and electrical properties, production scalability and ability to introduce various characteristics to everyday materials widen the spectrum of the tasks conventional materials (e.g. polymers) have been designed for. As such, use of CNTs in polymer adhesives opens door to a wide range of functions adhesives are used for. This is surging in an era where traditional methods of joining such as bolting or mechanical fasteners are becoming partly replaced by fastener-less technologies (e.g. adhesive bonding) for lightness, integrity and multifunctionality. Moreover, there is an urgent need to a progressive transition to bonded joints for assembly and joining high performance composite parts e.g. wing and fuselage components^1^. However, adhesives are sensitive to variations in service and hygrothermal loads, and also surface preparations^2–6^. Mechanical performance of adhesives under peeling stresses (in Mode I fracture; opening) is also relatively low^7^. Furthermore, disassembly of bonded joints for periodic inspections of internal damage (e.g. adhesion failure) is another downside as non-destructive inspection (NDI) methods are currently limited for reliable detection of such failure when zero-thickness bond defects are present^1,7–10^.

Therefore, research in structural health monitoring and bond damage detection methods is essential in order to prevent catastrophic failures that may arise due to barely detectable instantaneous adhesive bond failure^5,8,11^. Various researches have been conducted for that detection^6,12–14^ but there is little research done on strain measurement within the elastic regime of adhesive performing in bonded joints prior to irreversible damage occurring either in adhesive bulk or at its interface with an adherend.

**Figure 1:**
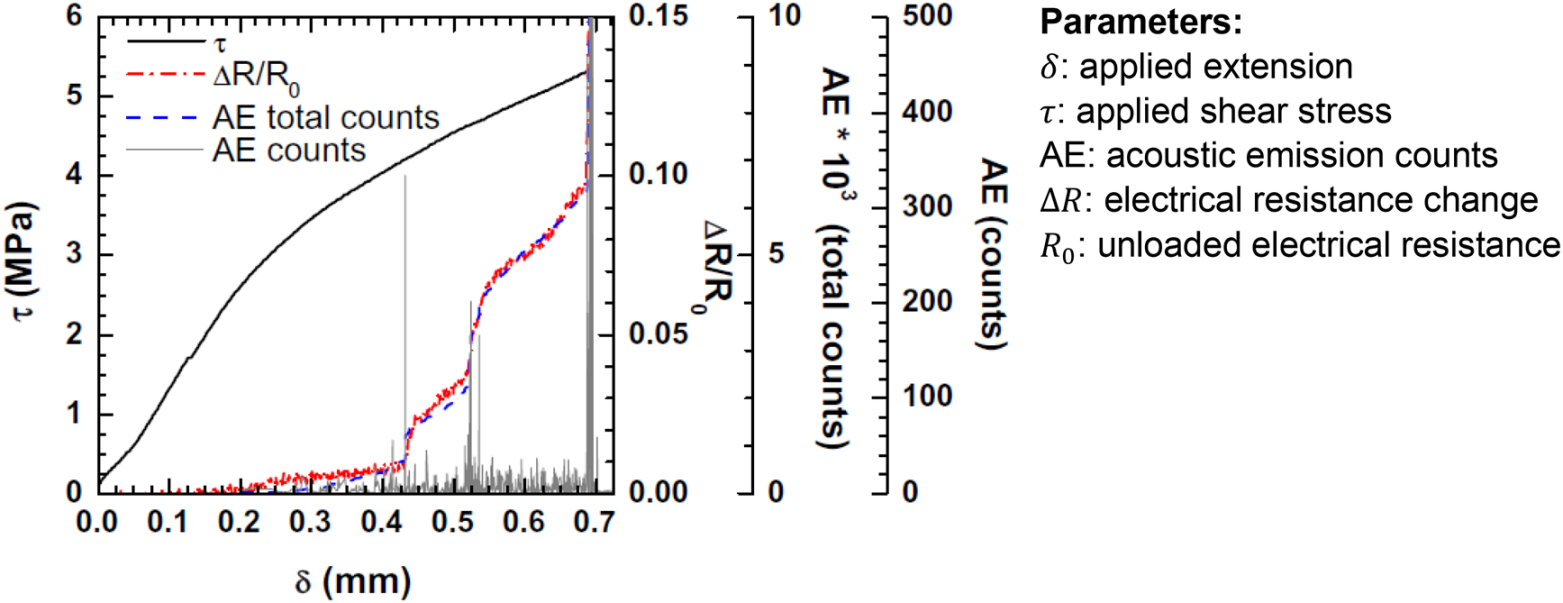
Utilisation of variations in ${\varvec{\Delta}}{\varvec{R}}/{{\varvec{R}}}_{0}$ trend for adhesion failure measurement^8^.

Numerous researches have focused on enabling strain sensing in polymers using functional nanomaterials including CNTs^8,15–23^ however there are a few researches conducted on the use of such technology for measurement of adhesion failure in adhesively bonded joint structures (e.g. aircraft wing’s leading edge). On the other hand, repeatability of the performance of the measurement technology, and therefore its reliability, within the elastic regime, rather than damage progression, e.g. in cyclic tension loading, has rarely been investigated. A research has examined single-lap bonded joints at different CNT fractions (0.5 wt%, 1.0 wt%, and 2.0 wt%) in epoxy adhesive^1^ relying upon the CNT’s piezo-resistivity. Though defects in polymer can be detected via enabling such CNT enhancement, the technology has not been able to identify bond defects in joints application^8^, and has partially been successful in detection of surface defects via resistance response under static loading^3^. Adhesion failure is an interfacial bond failure mechanism between the adhesive and the adherends, which is recognised by the absence of adhesive on one of the bonding surfaces. A distinct research in^8^ has shown that under monolithic tensile loading each failure mechanism possesses a distinct resistance response which corresponds directly to recorded acoustic events, as shown in Fig. 1. Though the size of such defect has not been quantified, the resistance response was able to detect the onset of damage long before the specimen reached its ultimate average shear strength, $\tau$, indicated by dramatic slope variations in the $\Delta R/{R}_{0}$ trend.

The current research develops a novel adhesion failure sensing technique using CNT modified epoxy adhesives. The article presents the underlying fundamentals behind the adhesion failure in high performance adhesively bonded joints, investigates the strain sensing capability induced by CNTs, at the onset and propagation of adhesive degradation, and identifies critical points during in-situ strain measurement to correlate with the joint performance in thick and thin bond thicknesses.

For the polymers enhanced by conductive nanoparticles, several distinct physical processes control the electron transport in the mixture. While the dispersion of nanoparticles does not allow direct mechanical contact between the particles, the electron transport is only possible through the polymer barrier^24–26^. In such case, the conduction between the CNTs is influenced by the quantum tunnelling barrier or tunnelling voltage resulting in interparticle tunnelling conduction between adjacent particles^27,28^, or by variable range hopping mechanism^29–31^. However, a long range microscopic conduction mechanism of electrons within a composite is governed by the percolation phenomenon^32,33^ with the most important element of the theory—percolation threshold, describing the minimum volume content of the conductive nanoparticle providing the electrical conductivity^34^. This phenomenon was deeply investigated for the carbon nanomaterials based composites over the past few decades, including CNTs^35–40^ and graphene platelets^41,42^, summarised by Bauhofer and Kovacs^43^. Likewise in our research, the CNT’s piezo-resistive effect—a change in the electrical resistance under mechanical strains^44^ (herein tensile cyclic strains)—is due to band-gap changes^45,46^ which represents an energy range in which no electron state can exist. Once CNTs are dispersed in a polymer, the composite becomes piezo-resistive not only because they are inherently piezo-resistive, also due to phenomenological reasons: when tensile strain is applied, contact between some CNTs is lost, and for some CNTs the resistance with neighbouring ones changes due to distance variations^28^.

## Fabrication of CNT modified adhesively bonded joints for strain measurement

### Design of performance testing of CNT modified bonded joints

According to the percolation theory, the electrical conductivity of the CNT modified adhesive is the function of the CNT volume, and thus given the Kirkpatrick’s equation^32^

1 $${\varvec{\sigma}}\approx {{\varvec{\sigma}}}_{0}{\left({\varvec{V}}-{{\varvec{V}}}_{{\varvec{c}}}\right)}^{{\varvec{t}}}$$

where $\sigma$ is the conductivity of the composite, ${\sigma }_{0}$ is the conductivity of CNT, $V$ is the volume concentration of the CNTs, ${V}_{c}$ is the critical volume of the percolation threshold, and $t$ is a critical exponent describing the type of conductive network. The equation implies that with the increasing $V$, $\sigma$ increases. While in the CNT modified adhesive used in the bonded joints the amount (and thus the volume) of the CNTs remains unchanged, and the overall volume of the joint (${V}_{J}$) decreases with the compression (either due to process control or during loading), the effective volume of CNTs calculated as ${V/V}_{c}$ increases, thus introducing a higher conductivity (lower resistivity). The sudden transition in the mixed CNT-epoxy (conductive-nonconductive) material from insulator to conductor occurs at the percolation threshold at which a connected network of sites is formed that spans the sample, causing the system to percolate^47^, and its value does not necessary have to be 100% of the matrix in which it’s been embedded, i.e. CNTs not covering the whole volume of the matrix material, but usually a relatively small fraction of the matrix volume above which the material becomes ‘conductive’. In that sense, the value of $V$ (i.e. the CNTs’ volume fraction) should reach ${V}_{c}$ for the material to percolate. The value of $\sigma$ is described by Eq. (1) at the vicinity of the percolation threshold (represented by ${V}_{c}$ herein). The equation is valid only for $V>{V}_{c}$, and the value of ${V}_{c}$ and $t$ is obtained from fitting a line to $\mathrm{log}(\sigma )$ data against the CNT fraction ($\mathrm{log}(V-{V}_{c})$) data. According to^47^, $\sigma$ dramatically increases above 4wt.%, presumably the percolation threshold according to the percolation theory, for the MWCNT-polymer system with an exponent of $t=$ 2.27% (nearly 2 according to the theory).

**Figure 2:**
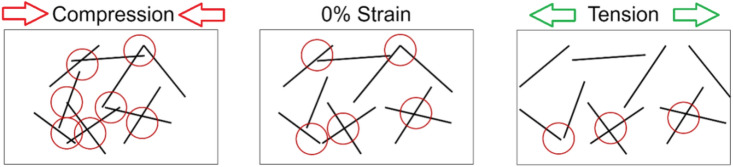
Evolution of number of conductive pathways (circles) between CNTs (solid lines) in polymer with mechanical load (compression and tension).

Moreover, assuming a constant CNT fraction and size, a thin adhesive should induce lower resistivity in a bonded joint than that bonded using a thick adhesive. This scenario has been designed in the current research to enable comparison of bond thickness effects on piezo-resistivity and sensitivity. Generally, the CNTs are pushed back against each other under compression, make more number of contact points (dashed circles in Fig. 2), which increase the conductivity and reduce the resistivity. An opposite effect can be observed when tension load is applied.

Inevitably in our case, the piezo-resistivity of CNT modified polymer is originated from intrinsic conductivity of single CNTs and the resistivity change of CNTs conductive network during mechanical deformation, also a function of the CNT’s chirality^48^.

**Figure 3:**
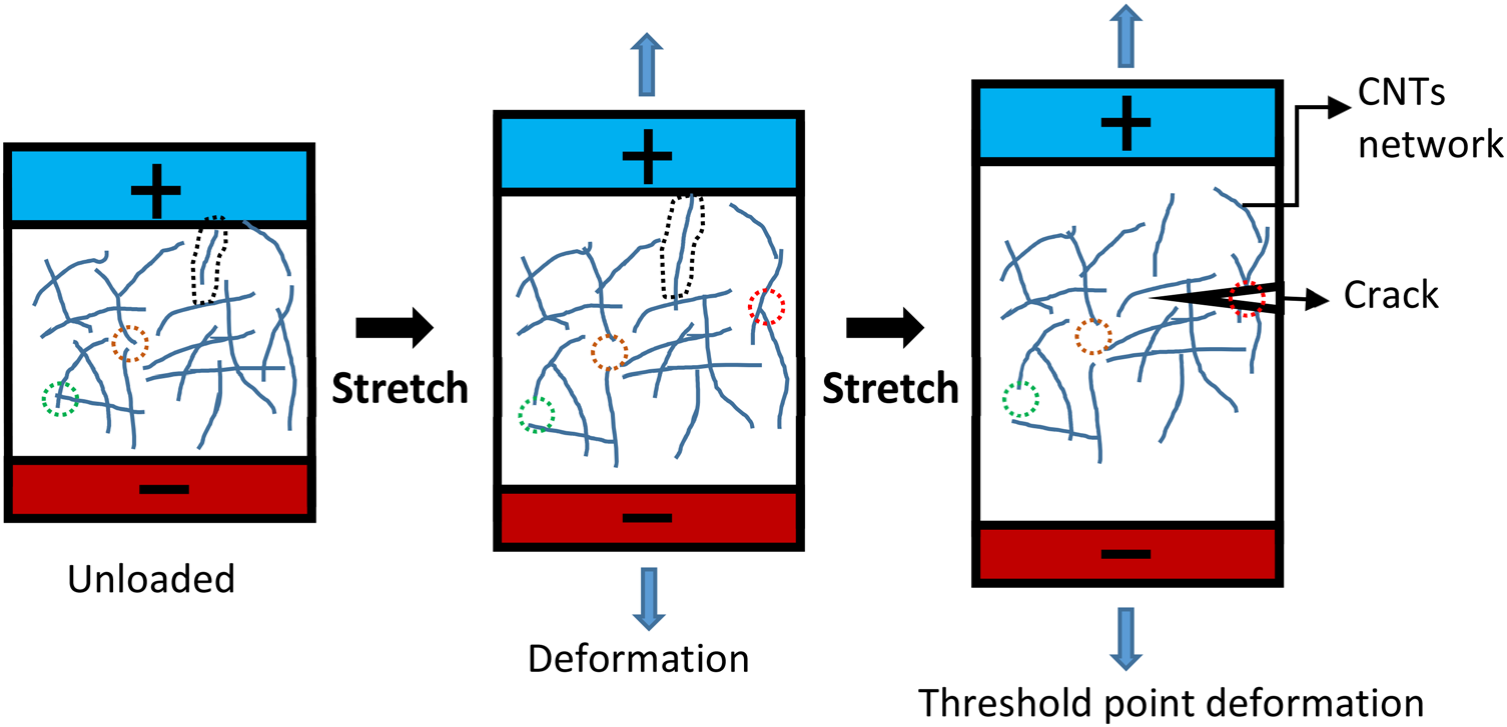
Schematic illustration of adhesion cracking at threshold point deformation inducing dramatic resistivity change (dashed circles indicate separation or elongation of three individual CNTs).

However, the electromechanical properties of CNTs network is much more complicated due to the facts that there are uncertainties associated with the dispersion and distribution of CNTs in a polymer^49^, as schematically illustrated in Fig. 3. Once the composite is stretched, individual CNTs inside would be also elongated which activates the intrinsic resistivity change of individual CNTs. It has been reported that the intrinsic resistivity of CNTs increases considerably at a relatively small strain^50^. In the case examined herein, a precise control of dispersion and distribution is very unlikely, as is the case for many scaled-up manufacturing techniques. Therefore having CNT enriched and polymer enriched areas is possible. Such matters interfere with the resistivity data measured simultaneously in real-time, and give unreliable indication of strain evolution, thus of damage evolution. An interpretation technique has been pursued in this research that proposes reliable measurements independent of such inevitable uncertainties induced inevitably during fabrication of the composite, described in “Results and discussion” section.

Also, elongation will separate the interconnection of adjacent CNTs and result in loss of CNTs contact and tunnelling points, which increase the material’s resistivity. At large deformation, micro-cracks may become present which either breaks individual CNTs or pull them out of the polymer (Fig. 3), thus increasing resistivity. It should be noted that such mechanism is irreversible meaning that the resistivity due to micro-cracking is not recovered after unloading^48–51^. Therefore, a resistivity baseline has been predicted in a loading–unloading cyclic scenario in our experimentation in which the applied stress exceeds the CNT-polymer bond strength, and the crack induced resistivity may not reversed back to the initial level. This phenomenon is utilised as a signal for the initiation of crack. In adhesively bonded joints, this states that either the bulk of adhesive cracks open or the disbond occurs at the adhesive-adherend interface. In a case that the adhesion strength is weakened (e.g. via poor surface preparation), the disbond scenario is more likely to occur. This has been designated using a peel ply process to draw the focus of localised high stress at the interface to ensure the adhesion failure would be dominant rather than bulk damage.

As final part of the experimentation design, there should be a certain point in an increasing loading scenario, at which the slope of the resistivity would dramatically be changed, indicating adhesion failure mechanism, and such point should be achieved no matter of non-uniform distribution of CNTs, a ‘threshold point’. This has been exploited in the current research for adhesion failure monitoring. Therefore, a load-unload cyclic tension test has been designed to examine such multi-parameter hypothesis outlined above.

### Selection criteria for efficient CNT modified polymer mixture

A number of researches have been conducted for development of piezo-resistive strain sensors made by CNT embedded polymers^52–55^, in which a higher sensitivity has been observed than that of conventional strain gauges, suggesting a promising measurement technology. The piezo-resistivity of such sensor is defined as $\Delta R/{R}_{0}$, where ${R}_{0}$ represents the original resistivity at zero strain (undeformed material), and $\Delta R$ the change of that at a certain strain $\varepsilon$^44^. The sensitivity of a sensor is then defined with the gauge factor $k$:

2 $$k = \frac{{{\raise0.7ex\hbox{${\Delta R}$} \!\mathord{\left/ {\vphantom {{\Delta R} {R_{0} }}}\right.\kern-\nulldelimiterspace} \!\lower0.7ex\hbox{${R_{0} }$}}}}{\varepsilon }$$

Such studies have used either a two-probe^56,57^ or a four-probe method^53,58^ for measuring resistance changes. They have identified that the piezo-resistivity of CNT modified polymers is approximately linear at low strains (e.g. within the elastic regime) and becomes non-linear at high strains^53,54,56,59^ under tensile strains, mainly due to the tunnelling resistance increasing non-linearly^44^. Also, percolation theory indicates that the conductivity change is nonlinear due to high probability of conductive paths formation when more CNTs are added. However the sensitivity could also be negatively affected at excess CNTs. With abundant CNTs, the beginning of deformation could cause negligible resistivity change, which means, the strain sensing property is weakened^43^. Therefore, a percolation threshold of CNT content is necessary for theoretical establishment of an efficient conductive network for piezo-resistivity. In other word, insufficient CNTs content in composites could degrade the sensing performance, and finding a proper content/concentration of CNTs is therefore vital to best optimise the overall strain sensing performance.

Here is one model for percolation threshold calculation in CNTs dispersion, which is given by^60^:

3 $$\phi_{c} = \frac{{\xi \varepsilon_{L} \pi }}{6} + \frac{{\left( {1 - \xi } \right)21.195}}{{\alpha^{2} }}$$

where $\phi_{c}$ is the actual percolation threshold, $\xi$ is the volume fraction of composite with agglomerated CNTs in case of non-uniform dispersion, $\varepsilon_{L}$ is the localised volume content fraction of CNTs in an agglomerate state, and $\alpha$ is the ratio of average length of CNTs over average diameter, i.e. $\alpha = l/d$. For our CNTs with average diameter of 60 nm and length of 35 micron, $\alpha$ = 583.3.

$\xi$ = 5% means that 5% volume of the composite is occupied by agglomerated CNTs. Also, $\varepsilon_{{\text{L}}}$ = 10% may mean that the CNTs occupy 5% of the volume in the hypothetical spherical shaped agglomeration. Then $\phi_{C}$ for such values is given as

4 $$\phi_{c} = \frac{{\xi \varepsilon_{L} \pi }}{6} + \frac{{\left( {1 - \xi } \right)21.195}}{{\alpha^{2} }} = 0.268\%$$

In practice, the CNTs quality depends on synthesis process and parameters, which could affect the average CNTs aspect ratio. Also, approaches like sonication, shear mixing, surfactant can improve CNTs dispersion, thus CNTs agglomeration could not be completely evitable^61^. Aspect ratio and agglomeration state could be roughly estimated with the help of optical microscopy and scanning electron microscope, and then a percolation threshold could be found in correlation with the aspect ratio and agglomeration state. This approach was taken in our research to induce a positive effect on finding suitable CNTs concentration and optimising strain sensing properties, described in “CNT modified adhesive and single-lap bonded joint test setup” section.

## Materials

### Adhesive

The CNT modified epoxy adhesive is a high strength aerospace grade 2-component epoxy-based thermoset adhesive containing multi-walled CNTs (average diameter of 100 nm) produced by chemical vapour deposition technique, both supplied by Cametics Ltd. Component A of the adhesive is an epoxy resin containing CNTs and component B is an amine curative, also containing CNTs before it’s mixed. The adhesive’s preparation for bonding has been described in “CNT modified adhesive specimen manufacture” section, and its response (mechanical and electrical) have been assessed in this research, and is described in “Single-lap bonded joint manufacture” section.

### Adherends

**Figure 4:**
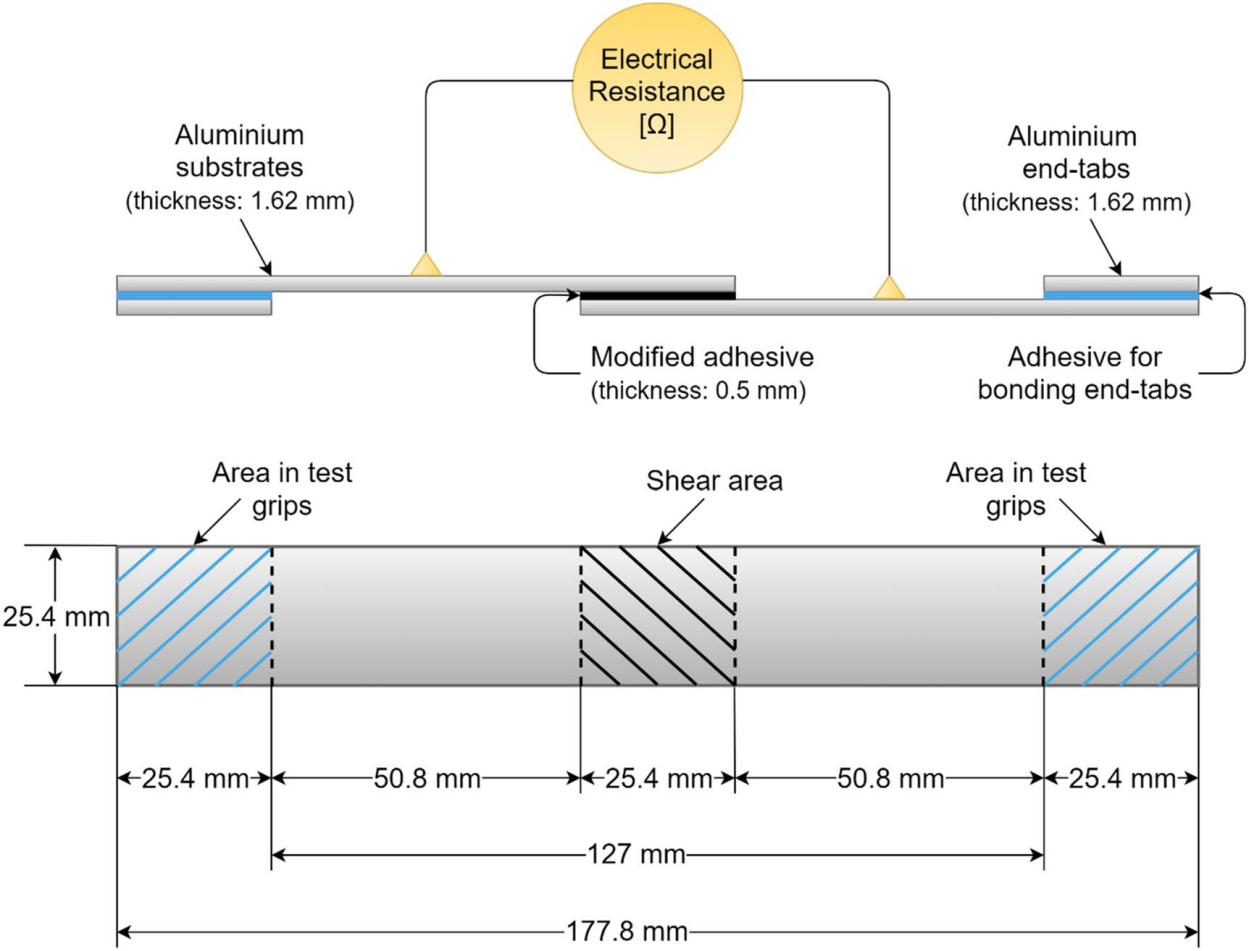
Single-lap bonded joint specimen and in-situ electrical measurement attachment.

The substrates used as adherends for fabrication of single-lap bonded joint specimens were aerospace grade aluminium 6063T6 with thickness of 1.62 mm, a medium strength alloy^62^. Aluminium as a material was chosen due to its high electrical conductivity, accessibility and straightforward surface preparation for bonding. Moreover, the failure strain and the yield strain for Aluminium significantly differs from those for the thermoset adhesive and the modified adhesive. Therefore, the resistivity change due to strains variations in the adhesive prevails in such disparity with Aluminium, meaning that the strain in adherends are negligible compared to that developing in the bond. Aluminium substrates were cut according to the dimensions based on the standard ASTM D1002 to manufacture single-lap joints as presented in Fig. 4, and were drilled at equal distances with the bond edges for electrical connections so as to be tested in situ for electrical resistance measurements. Electrodes with attached ring terminals were then bolted on, using M3 Hex socket bolts, and nuts with M3 washers to ensure a tightened connection and minimal effect on the strain data.

## CNT modified adhesive specimen manufacture

### Adhesive cure

**Figure 5:**
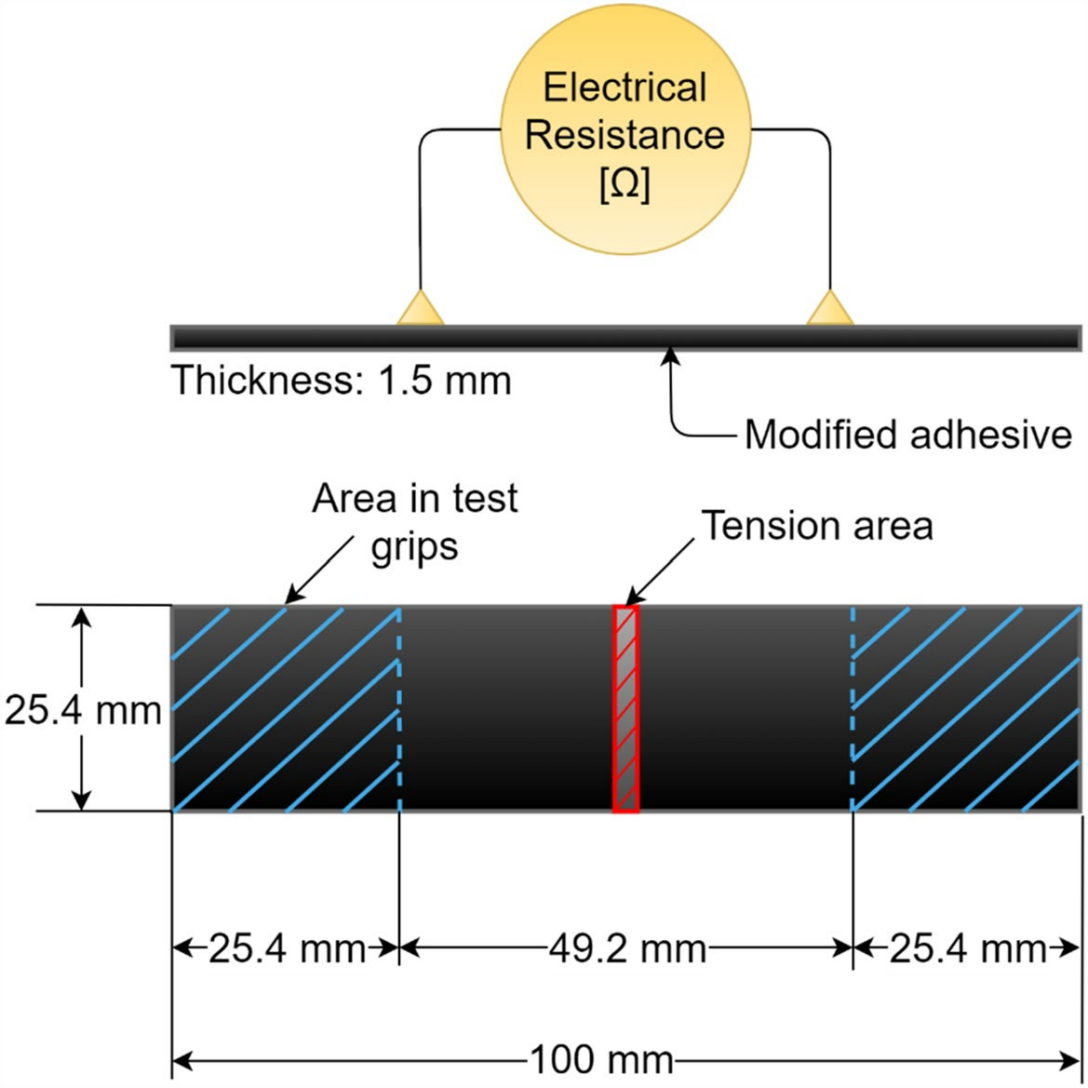
MWCNT modified adhesive specimen.

To examine the electrical and mechanical properties of the CNT modified adhesive independent of its use as adhesive in the bonded joints, adhesive specimens (Fig. 5) were fabricated having controlled thickness of approximately 1.5 mm in a steel fixture using spacers of 1.5 mm thickness. The adhesive specimens were then cured at 110 °C for 5 h. The specimens were measured post cure with micrometer, and the final thickness of each joint was identified.

### Cutting

Once cured, the steel fixture was disassembled, and the sheet of the adhesive was removed. The sheet was then cut into strips of the adhesive specimens to the dimensions presented in Fig. 5.

### Electrical insulation and electrode connection

**Figure 6:**
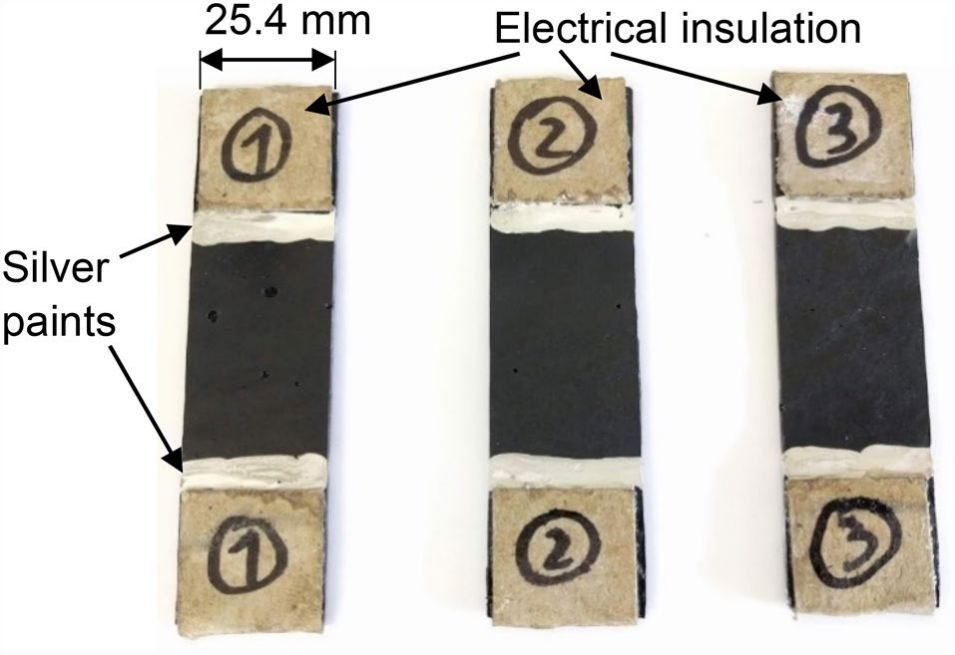
CNT modified adhesive specimens with bonded Kraft paper insulation.

Kraft paper was bonded using Cyanoacrylate-based commercial adhesive on the test grip areas of the strips to electrically insulate the adhesive specimens from the tensile machine grips during testing (Fig. 6). Electrodes with crocodile clips attached onto their ends were then clipped onto the specimens for enabling connection with multimeter. For improving the electrical connection between crocodile clips and the adhesive, electrically conductive silver paint Electrodag 1415 was applied on the specimen’s edges, similarly conducted in^63^.

## Single-lap bonded joint manufacture

### Surface treatment of adherends

Based on the chemical nature of polymer bonds, requirements for their processing and wettability are specified to chemically activate the adherends’ bonding surfaces which has positive influence on the adhesion properties (e.g. adhesion strength), and treat the surfaces so as to prevent the formation of hydrated oxides. There are several proven processes for aluminium such as phosphoric acid anodizing (below 29 °C), grit blast and silane coupling agent, and sol–gel process. The ends of the adherends, which represent the overlap region of the final specimens, were first abraded using 400-grit sandpaper, manually and perpendicular to the direction of loading (length of the adherends)^8^. This was done within one hour of adhesive application at room temperature and in a controlled laboratory environment to minimize any potential oxidation of aluminium's surface. Abraded surfaces were then wiped with paper towels soaked in acetone, and were additionally rinsed with acetone to remove any leftover contaminants, recommended by researches in^1–3,64^. The joints were tested not longer than 24 h after their manufacture.

### Fixture preparation

**Figure 7:**
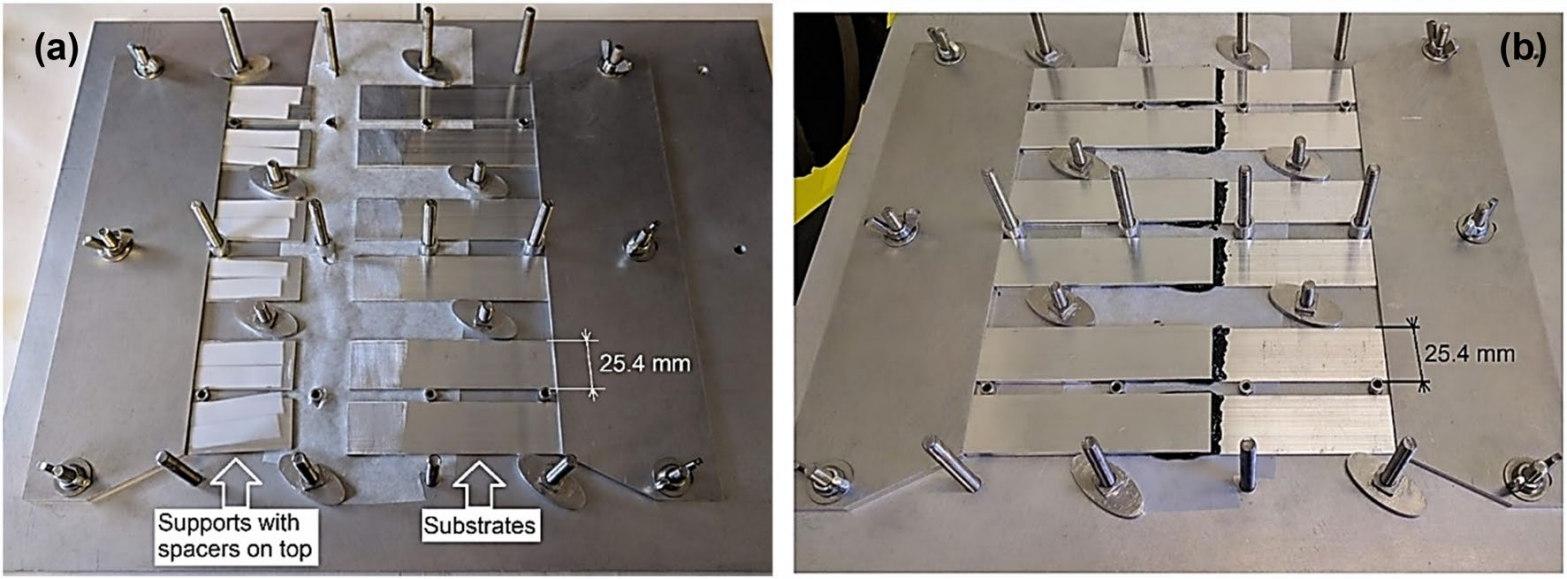
Fixture preparation: (**a**) positioning of bottom adherends and supports with 0.5 mm-thickness Teflon spacers, (**b**) positioning of upper adherends and pressurisation.

**Figure 8:**
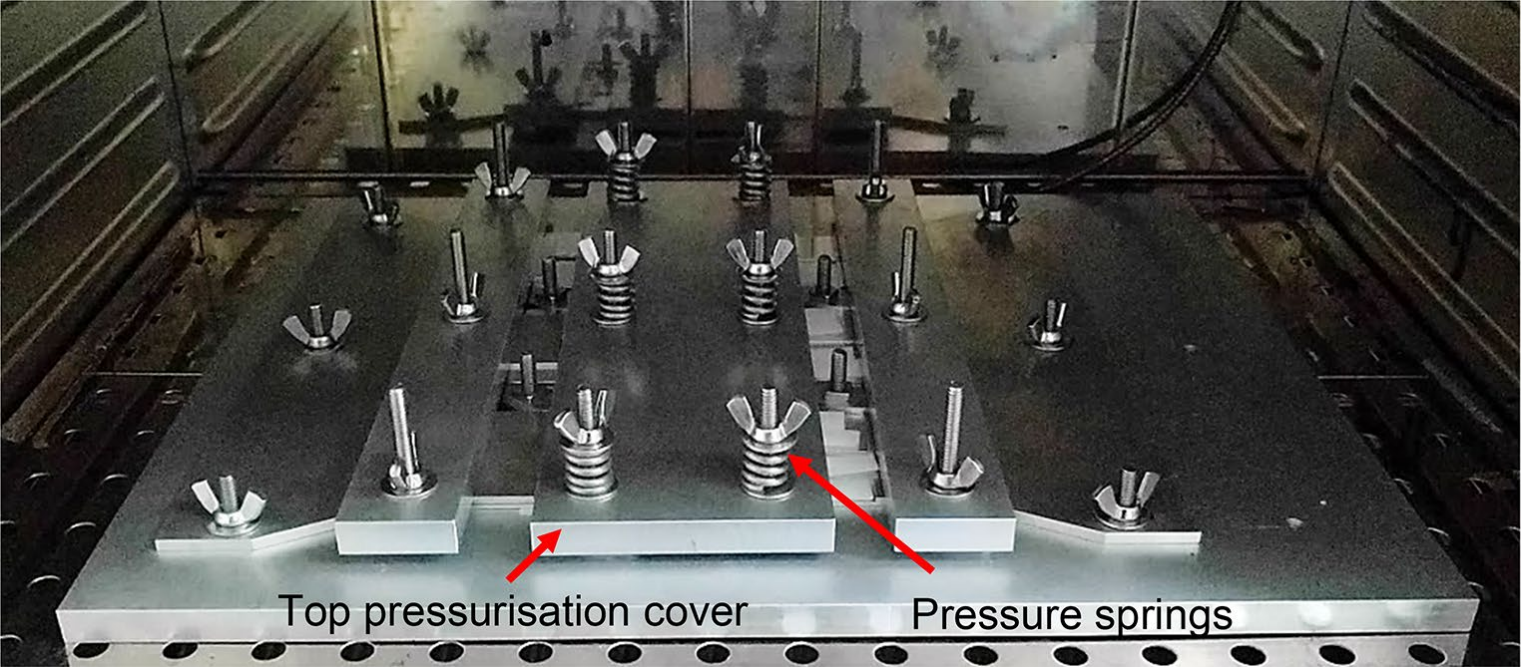
Curing of adhesively bonded single-lap joints in heating oven.

A fixture to assemble the single-lap bonded joint specimens was manufactured and assembled as seen in Fig. 7. Oven paper was put in the middle of the fixture underneath the adherends (post treatment) to prevent the adhesive sticking to the fixture. Bottom adherends were then fixed in place with the treated surfaces (bond region of 25 mm × 25 mm) facing up (Fig. 7a). On their opposite side, aluminium supports were also fixed in, and standard 0.5 mm-thickness Teflon spacers were positioned on top to produce bond thickness of 0.5 mm slightly lower than the recommended thickness of 0.67 mm. The jig has not been optimal due to the existing tolerances which falls below 0.1 mm. As a result, additional supports were required on the left side of each pressure steel panel (left arrow in Fig. 8) to guarantee uniform pressure to acquire correct and uniform bond thicknesses across all samples.

### Adhesive preparation and application

The adhesive was prepared in a fume cupboard by stir-mixing its two parts in a weight ratio of A:B equal to 2:1. The two parts were weighed to the nearest tenth of a gram using Ohaus Scout Pro Portable Balance scale, where part A was added first, followed by part B addition. Using a disposable spatula and a disposable container the product was stir-mixed for three minutes, scraping the sides and bottom of the vessel frequently.

Post mixing, the adhesive was slowly applied onto the substrate’s treated surfaces with a spatula over the bond region of 25 mm × 25 mm. The application was done as evenly as possible to minimize entrapment of air after positioning upper substrates in following steps.

The upper adherends were then positioned on top under an angle to enable an even distribution of adhesive and to push out the entrapped air (see Fig. 7b). After ensuring all supports were in place, the cover plates were positioned on top of the upper adherends. To ensure proper bond thickness, mechanical pressure was applied onto the plates through springs, which were tightened to a spring height of 16 mm. This was 178 N of force per spring, which causes the following pressure on each adhesive bond, as recommended by the ASTM Standard:

5 $$P = \frac{nF}{{iA}} = \frac{{6 \times 178.0\,{\text{N}}}}{{6 \times 25.4\,{\text{mm}} \times 25.4\,{\text{mm}}}} = 0.28\,{\text{MPa}}$$

where $P$ is the *a*pplied pressure on bond, $n$ the number of springs, $i$ the number of specimens (bonds), $F$ is force value per spring, and $A$ is the bond’s overlap area. The calculated pressurisation level was achieved using the springs at top of each support. The application was conducted manually, i.e. via turning nuts on top of each spring. The spacers to ensure nominal 0.5 mm bond thickness were made of relatively hard plastic, so they could have been pressurised. The effect of pressurisation directly on the spacers was not measured, but indirectly via measurement of the bond thickness achieved post assembly.

The fixture was then put into a pre-heated oven for adhesive to be cured at 110 °C for 5 h (Fig. 8). Differential scanning calorimetry was conducted on the adhesive post cure to ensure that the adhesive (CNT modified and unmodified) is fully cured.

Once the specimens were cured, they were removed from the fixture and carefully trimmed manually using sand papers to remove residuary side fillets of the adhesive joints, as seen in^2,3^, and to mitigate disparity in results due to the presence of such fillets.

### Tabbing

**Figure 9:**
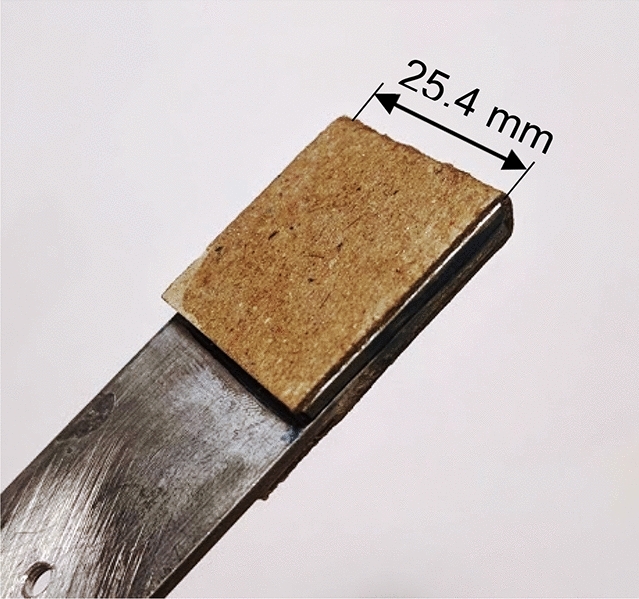
End tabs covered by electrical insulation (width = 25.4 mm).

End-tabs cut from the aluminium panel were approximately 25.4 mm × 25.4 mm × 1.62 mm (Fig. 9). For bonding tabs to the specimens, a two-component Araldite 420 A/B epoxy adhesive was used, which features high lap-shear and peel strength. After applying the adhesive and positioning the tabs, the tabs’ bond was cured at 60 °C for 12 h (ensuring below the glass temperature of the CNT modified adhesive). To prevent the tabs from slipping during curing, binder clips were used to hold them on, and to apply additional pressure to the joint.

### Electrical insulation

The specimens were electrically insulated in the gripping areas of tensile machine Instron 5500R 6025 equipped with a MERLIN software (https://www.merlinco.co.uk/products/merlin-survey-software/) in order to eliminate any interference in the electrical resistance measurements (Fig. 9), as emphasised in^57,65^. Preliminary trials were performed to assure that no electrical short circuit with the tensile machine grips occurs. The ends of the specimens were insulated via bonding strips of Kraft paper onto them. The adhesive was additionally spread on top of the paper and got soaked in, for further stiffening the Kraft paper.

## CNT modified adhesive and single-lap bonded joint test setup

### Consideration of joint eccentricity

**Figure 10:**
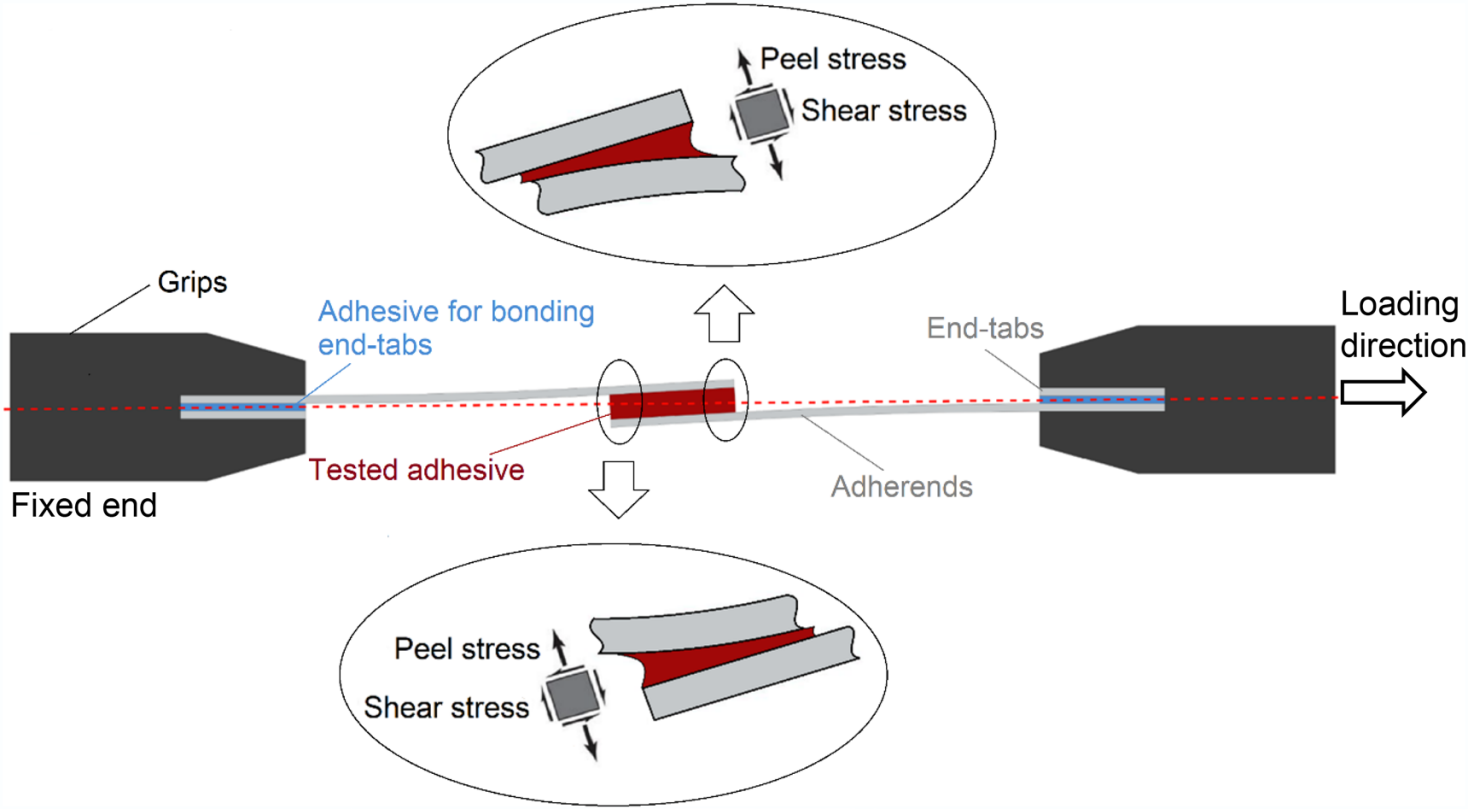
Eccentricity phenomenon in a single-lap adhesively bonded joint and consequential dominant peeling stress.

Quasi-static and cyclic mechanical tensile tests were conducted on the adhesively bonded single-lap specimens according to ASTM D1002–10, followed in some researches^1–3,64^*.* The most dominant stress present in single-lap bonded joints is peeling stress^66,67^ which is unevenly applied throughout the bond length due to out-of-plane bending moments caused by the eccentricity of the load path in such unsymmetrical joint^68^, as seen in Fig. 10. This results in peeling stress driven failure for an adhesive bond in single-lap joints, which implies that the single-lap joints provide conservative failure load prediction^6,12^.

To reduce the eccentricity, end-tabs were bonded to the adherends. However, due to the presence of variability in the thickness of adhesive bonds (of both adherends and end-tabs), complete elimination of eccentricity in a single-lap joint is not possible. A representation of this phenomenon is schematically depicted in Fig. 10 where the thickness of the CNT modified adhesive (nominal 0.50 mm) is exaggerated to be much higher than the thickness of adhesive at the end tabs (nominal 0.12 mm).

The specimens have been tested under the static loads in tension associated with in situ electrical resistance measurements described in the following section.

### In-situ electrical resistance measurement under mechanical loading

#### Equipment

An Instron 5500R 6025 electro-mechanical machine was utilised for static and cyclic tensile testing, instrumented by a calibrated 5kN load cell, LVDT and data acquisition system; however, the LVDT displacement measurements is not precise since it cannot compensate for the compliance of the crosshead, load cell and grips that come in the load. Therefore, a non-contact laser extensometer model (Epsilon LE-05) was used for displacement measurements.

For the electrical resistance changes (${\Delta }R$ measurement), a LabVIEW programme (National Instruments LabVIEW 2015; https://www.ni.com/en-gb.html) was developed and associated with the acquisition system having minimum resolution of 90 measurement per minute. A Keithley Multimeter 2000 with a Keithley 2000-SCAN Scanner Card was used for resistance changes measurement with a two-probe method. National Instruments CompactDAQ with CNI-9215 C Series Voltage Input Module was used to acquire load and extension signals from the Instron machine, and also to acquire the more precisely calibrated extension measurements from the laser extensometer.

#### Measurement algorithm

**Figure 11:**
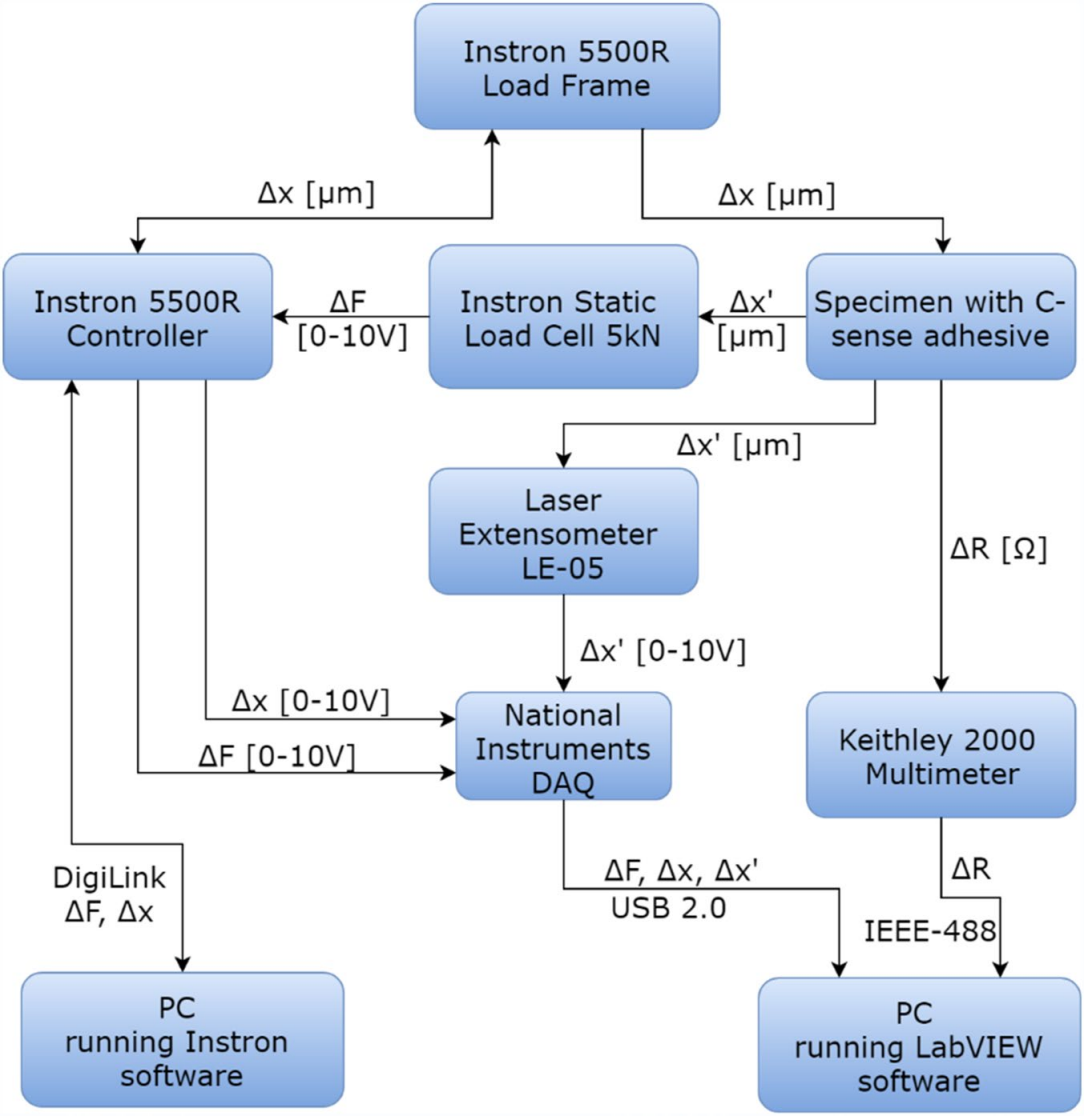
Iterative in-situ measurement algorithm for LABVIEW program.

Figure 11 shows the measurement algorithm developed for the testing and data acquisition, described in detail here:

Upon starting a test, the Instron software sends a command to the controller through the DigiLink interface requesting a particular change in crosshead position, Δx. The controller, in turn, sends the command signal to the load frame, and the crosshead changes position for an increment of Δx. The specimen which is fixed in the grips elongates for the same increment Δx; however, because of the interfering compliance of load-path components, the actual extension, Δx’, attain a slightly lower value than the command one. Consequently, the load-cell measures an increase in tensile load and sends an analog signal (in the range of 0–10 V) to the controller. The controller also receives a confirmation about the position change of the crosshead from the load frame.

In parallel, a laser extensometer measures the precise extension value of Δx’, and sends the data to the National Instrument DAQ system, which also receives analog data about the change in load, ΔF, and the crosshead position from the controller. Resistance measurements on the tested specimen are then performed by the Keithley Multimeter. The data from the National Instruments DAQ and Keithley Multimeter are digitally sent through IEEE-488 and USB 2.0 cables to a separate PC with the LabVIEW software developed in this research, where they are displayed and saved. Data for ΔF and Δx are also sent back to the Instron PC where they are displayed and saved. PC then increases the crosshead position to a new increment, sends the command to the controller, and the aforementioned process repeats.

#### Testing procedure for CNT modified adhesive only specimens

**Figure 12:**
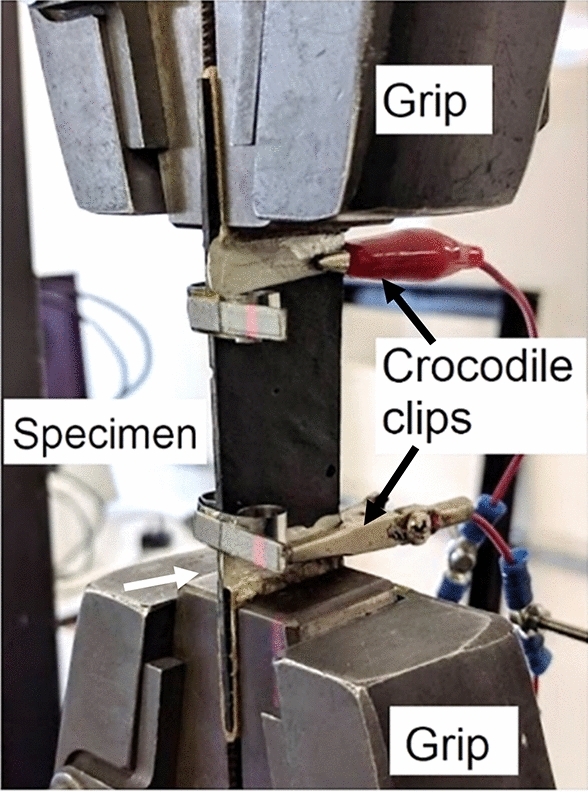
CNT modified adhesive specimens set-up in tensile machine’s grips equipped with laser extensometer.

The same equipment as for the joint specimens (described below) was used for testing adhesive specimens. Each adhesive specimen was gripped into a universal tensile machine equipped with a load-cell and laser extensometer, as shown in Fig. 12. Electrodes were then attached through crocodile clips onto the areas covered with silver paint. Reflective targets for the laser extensometer were clipped on at the edges of applied silver paint to enable comparison between data from the two instruments.

#### Testing procedure for single-lap bonded joint specimens

**Figure 13:**
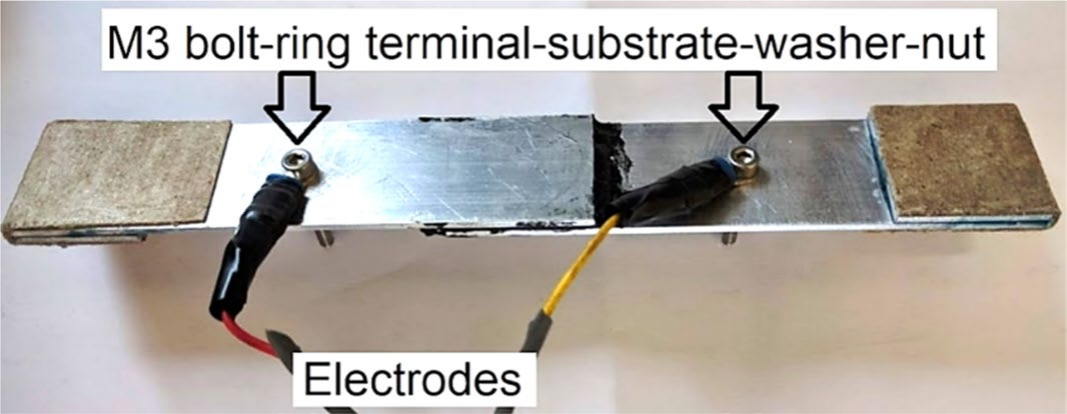
Electrical connection of electrodes on the aluminium adherends in single-lap adhesively bonded joints.

Both quasi-static and cyclic tensile tests were conducted in a temperature-controlled laboratory space at room temperature. The specimen’s electrode connection areas were slightly sanded manually and using 400-grit sand paper. Electrodes with attached ring terminals were then bolted on using M3 bolts, nuts and washers, shown in Fig. 13.

**Figure 14:**
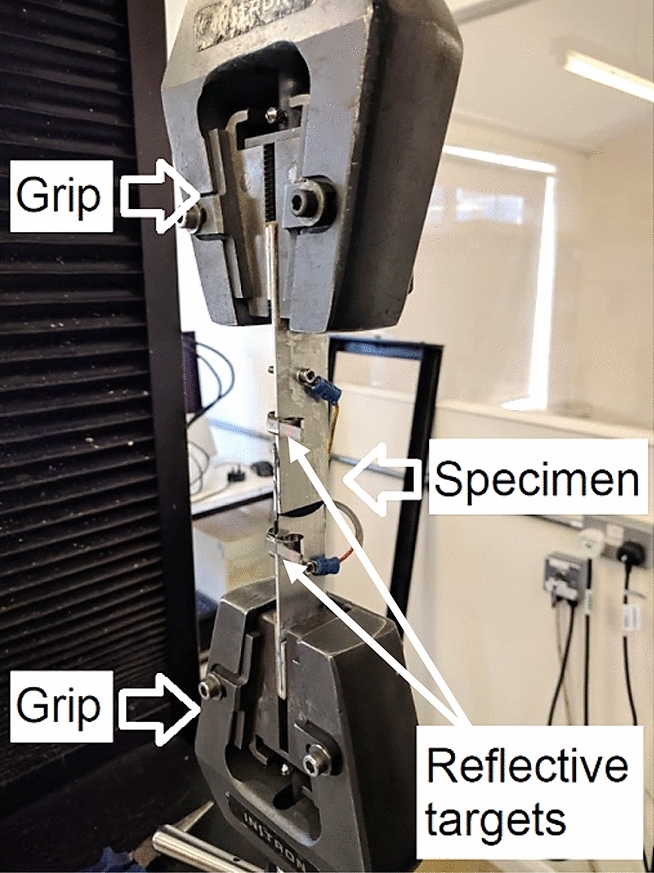
Single-lap bonded joint specimens set-up in test machine’s grips equipped with laser extensometer.

The specimens were then fixed into wedge-action grips of a tensile machine, and reflective targets for the laser extensometer were clipped onto the sides of each, as shown in Fig. 14. The two targets were positioned approximately 10 mm away from the top and bottom edge of the overlap area to be as close as possible to the failure mechanism without interfering with it.

The laser extensometer was then positioned at a pre-defined distance from the targets under a slight angle ($<$ 5°) to avoid possible spurious reflections from other reflective surfaces. Crosshead speed (0.42 mm/min) of the tensile machine was then set. Because the Keithley Multimeter had a relatively slow sampling rate of 1.5 measurements per second, it was decided to use a lower crosshead speed in comparison to the ASTM D1002 standard value of 1.27 mm/min to gather more data.

In the case of incremental cyclic tensile tests, where the force is set to be zero before introducing the next strain level^69^, the load increment was then selected, and set to be 300 N. The selection of this increment was based on the existing research in^16,64,65^ and according to the ultimate failure load in quasi-static and monotonically loaded specimens, so that the samples could achieve minimum three load-unloading loops before their ultimate failure.

## Results and discussion

### CNT modified adhesive’s quasi-static tensile test data

**Figure 15:**
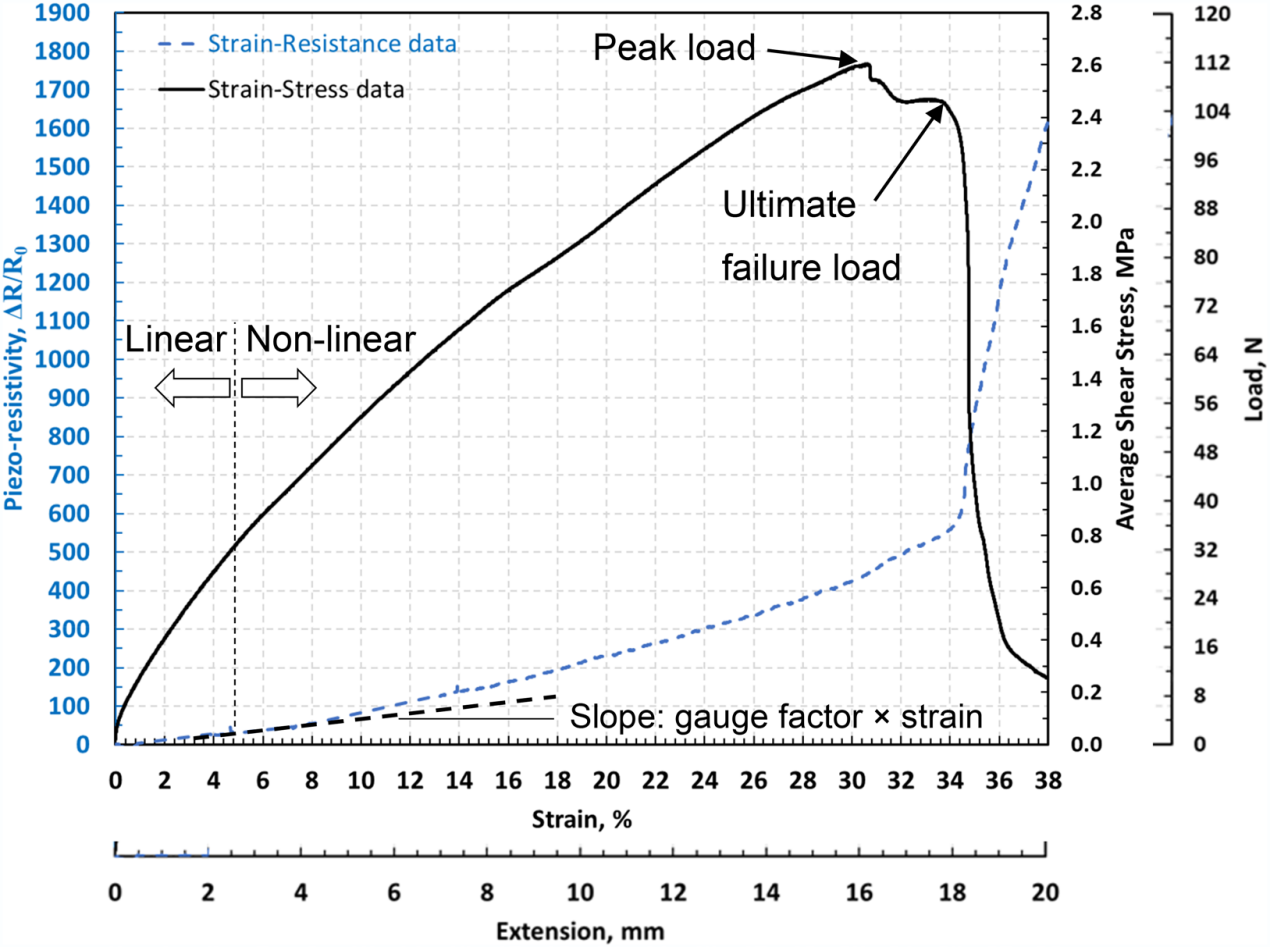
Evolution of ${\Delta }R/R_{0}$ response of CNT modified adhesive specimens during a quasi-static tensile test.

The CNT modified adhesive strips (Fig. 6) were manufactured under the same curing conditions as for the bond cure in the joint specimens, to characterise their electrical resistance response under identical conditions, subjected to tensile loads. The ${\Delta }R/R_{0}$ data was obtained under tensile loads and with respect to the laser extensometer data until the ultimate failure was reached (full cross-sectional net-tension failure), as shown in Fig. 15.

As seen, the initial linear piezo-resistivity region with a linear gain factor of 14.9 mm^−1^ (according to Eq. (1): $k =$ 735) was identified followed by a non-linear piezo-resistivity trend region until the peak load point (16.3 mm and 112.5 N) and the ultimate failure point (18.0 mm and 106.4 N). Once the peak load is reached, damage is developed across the area of the thin specimens which results in full breakage of the cross-section at the ultimate failure, and a sudden drop in load carrying capacity, which consequently leads to sudden increase in the resistivity due to the reduced area. The specimens exhibit slight ductile failure post ultimate failure attributed to the CNT bridging mechanisms in action.

The general trend of the non-linearity in the piezo-resistivity response is attributed to the reduction in conductive CNT paths and tunnelling as the CNTs start to separate under shear and tensile strain. Additionally, an indication of sensitivity of adhesive alone when it is loaded in tension was specified. From the slope of the linear region, the gauge factor obtained represents a sensitivity level more than six times higher than the sensitivity achieved with conventional strain gauges.

### Single-lap joints’ monotonic static data

**Figure 16:**
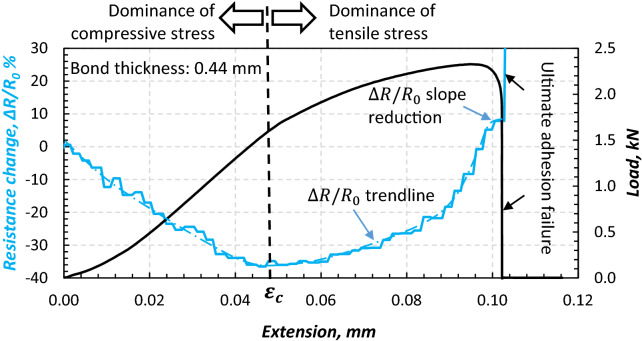
Evolution of ${\Delta }R/R_{0}$ in single-lap bonded joints with CNT modified adhesive during a quasi-static tension.

**Figure 17:**
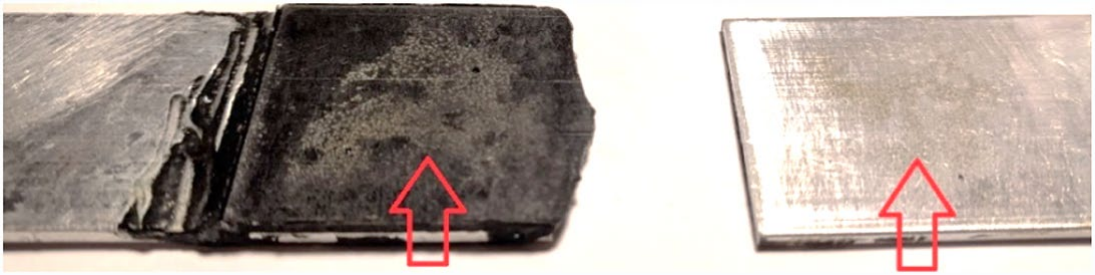
Ultimate adhesion failure in single-lap bonded joint specimen (arrows point bonded areas).

Four joint specimens were tested under static tension until their ultimate failure was reached. The load-extension data for one joint with the actual bond thickness of 0.44 mm is shown in Fig. 16. As seen, the ${\Delta }R/R_{0}$ decreases with the increasing load until a minimum negative value of 38 is reached at an extension of 0.048 mm and 1.6 kN, denoted by $\varepsilon_{c}$. The trend is then changed for loading beyond that at point $\varepsilon_{c}$ until full bond failure is reached, dominantly and ultimately in adhesion failure mode (Fig. 17), a dominant mechanism observed for all joint specimens, i.e. only deformation mechanisms were observed in the bulk of the adhesive, and damage was present at the interface as adhesion failed.

The $\varepsilon_{c}$ at which ${\Delta }R/R_{0}$ trend changes is attributed to the bond thickness difference between the CNT modified adhesive and the Araldite adhesive used for bonding end tabs and its effect on the joint’s eccentricity.

**Figure 18:**
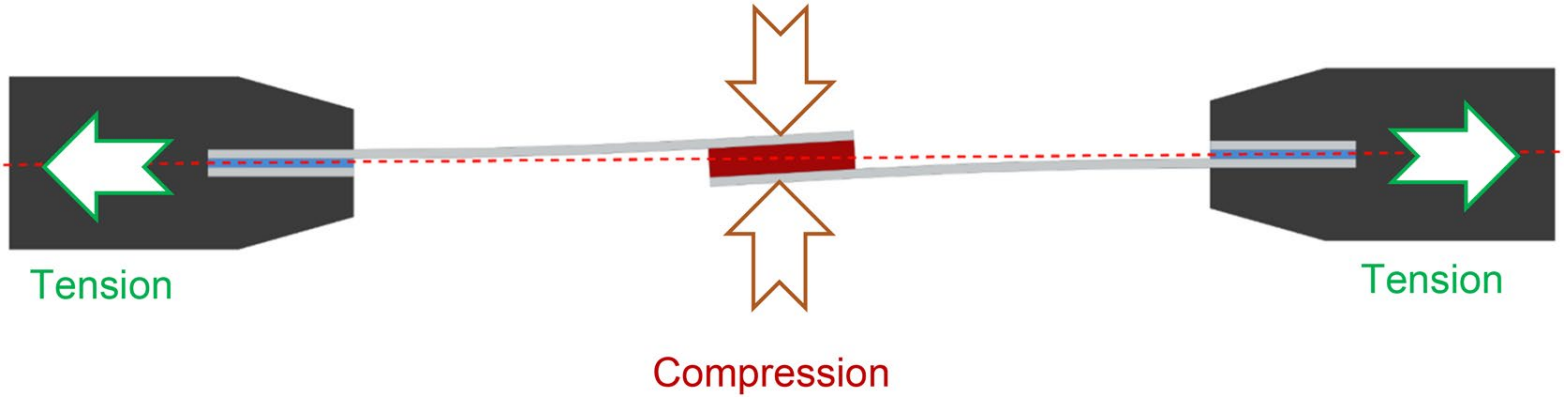
Eccentricity introduced by the bond thickness difference and consequent compression forces.

Thickness of the modified bond was averagely 0.47 mm while thickness of the Araldite bond was 0.12 mm. Eccentricity is not solely due to the bond thickness, and is inherent to single-lap joints, however the difference in bond thickness may additionally contribute to that: Once the specimen was fixed into the machine grips, compression traction forces were introduced to the joint perpendicular to the load axis (Fig. 18) occurring due to the single-lap adherend offset, in which the higher the bond thickness the higher the compression. Such compression at the initial phase of loading leads to compressive stress through the thickness of the bond (note that the compressive stress was also observed during clamping the specimens prior to loading.). The compressive stress then slightly reduces the thickness (as result of through-the-thickness compressive strains) that leads to increased conductivity due to increase in CNT contact points and reduced ${\Delta }R/R_{0}$. Upon longitudinal loading, once the joint is sufficiently tensioned, shear and tensile stresses reduce CNTs contact points, reduce conductivity, and increase resistivity, and at $\varepsilon_{c}$ reduce the dominance of the compressive stress resulting in increase in ${\Delta }R/R_{0}$.

During extensive tensile loading of the joint (close to the ultimate failure point), the CNTs become aligned and inclined towards the loading direction, which results in the conductivity increase despite the reduction of contact points. This might have attributed to the drop in the ${\Delta }R/R_{0}$ slope near the ultimate failure point in Fig. 16 (at 0.1 mm extension).

**Figure 19:**
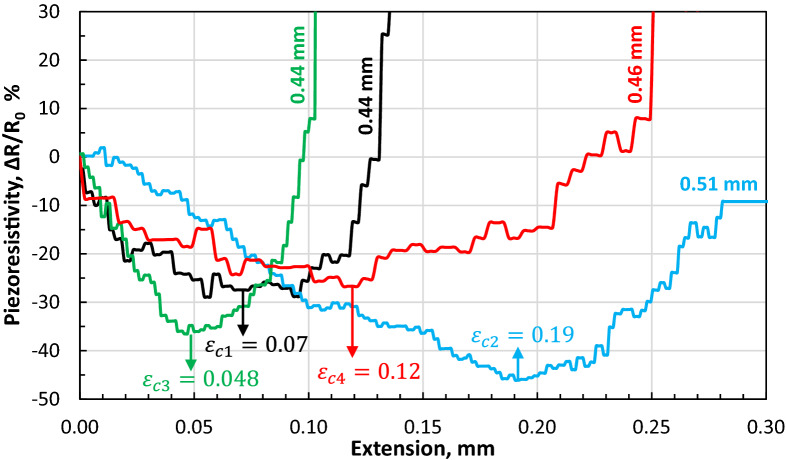
The evolution of ${\Delta }R/R_{0}$ for four single-lap joint specimens bonded with the CNT modified adhesive at different bond thicknesses of 0.44, 0.44, 0.46 and 0.51 mm.

Simply put, the CNT modified adhesive’s piezo-resistivity under compressive strains is anti-symmetric compared to that under tensile strains, therefore the piezo-resistivity reaches at its minimum level at $\varepsilon_{c}$ in Fig. 16. At this point the CNTs are now being pulled apart which reduces the number of conductive contacts and the tunnelling effect between them. The ratio between through-the-thickness compressive and tensile stress playing an important role at the beginning of applied tensile (longitudinal) force is the important factor in the evaluation of the acquired results, identified by the identification of $\varepsilon_{c}$. It may be noteworthy that the direction of the compressive stress discussed herein is directed through the thickness of the bond (rather than its longitudinal direction), induced by the work of joint’s misalignment/eccentricity at the beginning stage of a longitudinally applied tension (shown in Figs. 10 and 18). Until the longitudinal $\varepsilon_{c}$ (induced by the tensile force) is reached, with the compressive stress dominating, the pushed-together CNTs within the polymer matrix decrease bond’s electrical resistance (i.e. increase in conductivity). Above $\varepsilon_{c}$, the through-the-thickness tensile stress dominates pulling the CNTs apart (exaggeratedly illustrated in Fig. 3)—a process co-assisted by the epoxy matrix’ lower tensile strength than its compressive one—which leads to increase in the electrical resistance of the bond, along with the increasing longitudinal $\varepsilon_{c}$. Before the bond failure, ${\Delta }R/R_{0}$ increased due to disbond at the adhesive-aluminium interface, as evident Fig. 17 and by the research conducted in^8^. With the intention of finding additional evidence to support this hypothesis, ${\Delta }R/R_{0}$ data of four joint specimens bonded with their modified adhesive causing disparity in bond thicknesses are plotted in Fig. 19.

One distinctive point is that the number of turns on the top nuts to achieve the calculated pressure during the joints assembly (“Adhesive preparation” section) was not necessarily led to reaching the spacers as such (~ 0.5 mm) in some samples, and exceeded that level in some samples via pressurising the spacers even more though slightly. Nevertheless in all samples, the polymer flooded off the joint overlap edges once the pressure was manually applied. Such uncertainties associated with the tolerances and the manual pressurisation process resulted in joints having bond thickness variations from the nominal 0.5 mm.

It is observed that with the increasing bond thickness of the modified adhesives in the joints even slightly, $\varepsilon_{c}$ increases. This complements the aforementioned hypothesis: At higher bond thickness (e.g. 0.51 mm) the eccentricity angle of the joint (Fig. 18) is at higher value at the beginning of the test (before shear and tensile stresses dominate). Consequently, higher compressive strains are present in relatively higher bond thicknesses, leading to a higher $\varepsilon_{c}$ (the case for 0.51 mm bond thickness in Fig. 19). This phenomenon was pursued during cyclic load testing as well to compare data between the thin and thick bonds in the bonded joints (discussed in “Performance of CNT modified bonded joints under cyclic loading” section).

It should be noted that the thickness of the Araldite adhesive was controlled via holding binder clips where a relatively thin adhesive was applied (approx. 0.1 mm as measured after assembly). No spacer was used for tabbing therefore such thin bond thickness was produced. The tab bond thickness was ¼ of the joints’ modified bond thickness at its maximum level (one sixth of the joints’ bond thickness at its minimum). The effect of such ratio on the eccentricity, and therefore on $\varepsilon_{c}$ variations, has been minimal. Ultimately, the effect on the sensing response of the modified adhesive has been considered negligible in our study. It should also be noted that tab bond thickness ratio (1/4–1/6 of the joints’ bond thickness) would have made an almost similar effect on the comparative results across all samples in Fig. 19, i.e. assuming non-negligible effects from the tab bonds, those effects have been almost identical for all curves.

**Figure 20:**
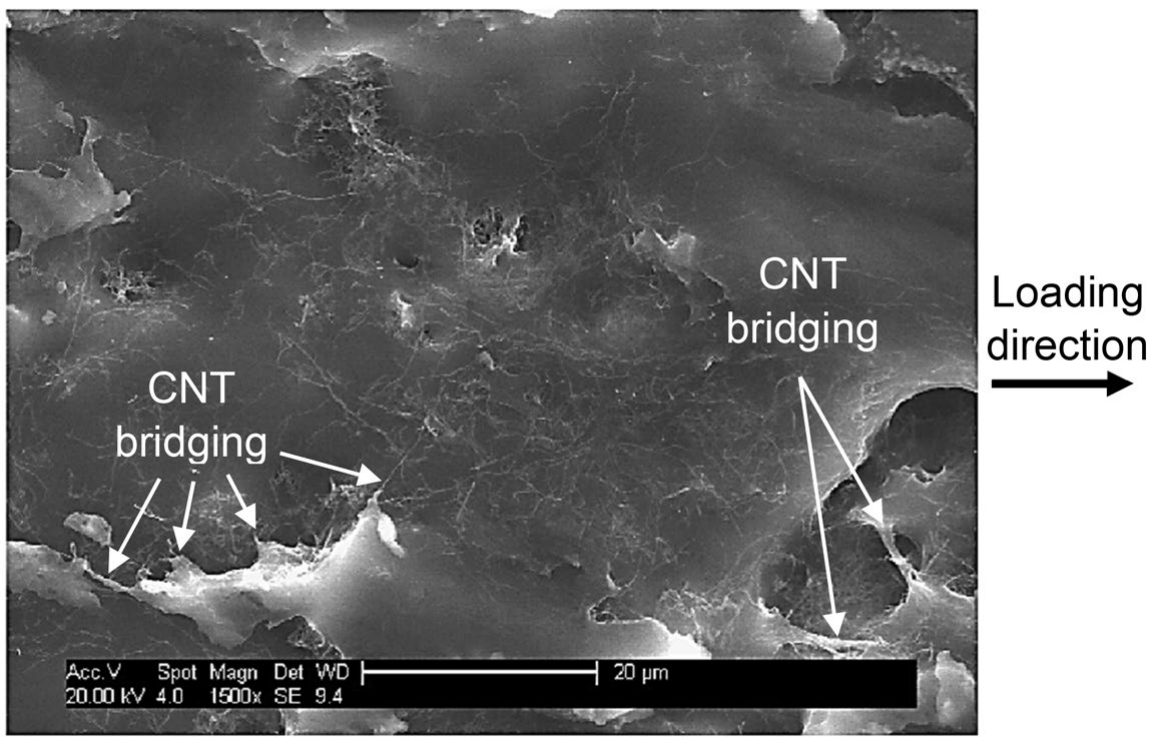
SEM image of single-lap joint specimen’s failure surface (Fig. 17) with CNTs dispersed in the polymer matrix.

**Figure 21:**
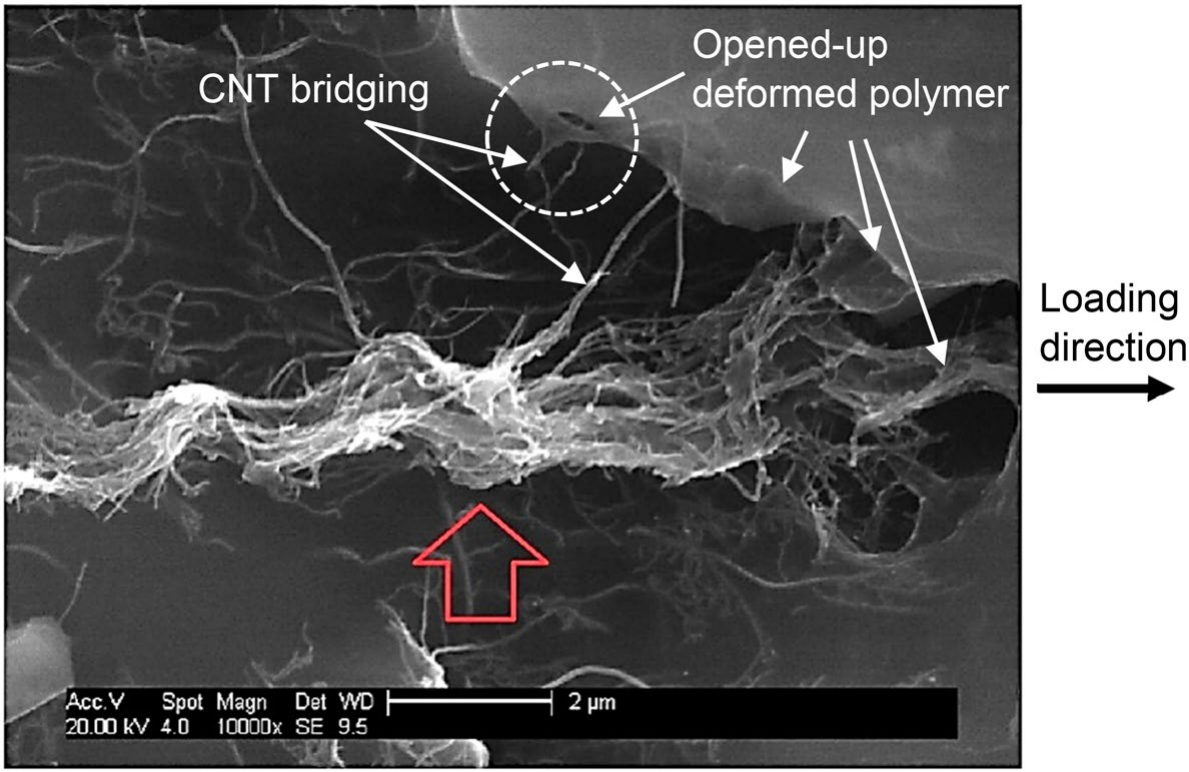
SEM image of agglomeration of CNTs with bridging between cracked open polymer matrix; image from bridging and cracked open polymer shown in dashed-line circle.

The SEM image of the CNT modified adhesive bond’s cross-section in one joint at ultimate failure is shown in Figs. 20 and 21, in which the CNTs deformation and their bridging mechanism within the highly deformed polymer material are evident, protruding out of the polymer surface.

In Fig. 21, typical agglomeration of CNTs is observed in a few locations across the failure surface, but more importantly the CNTs bridging mechanism between the cracked open deformed polymer materials. Both CNT clusters and the bridging mechanism at the adhesion surface are observed to be mostly occurring in the loading direction.

### Performance of CNT modified bonded joints under cyclic loading

**Figure 22:**
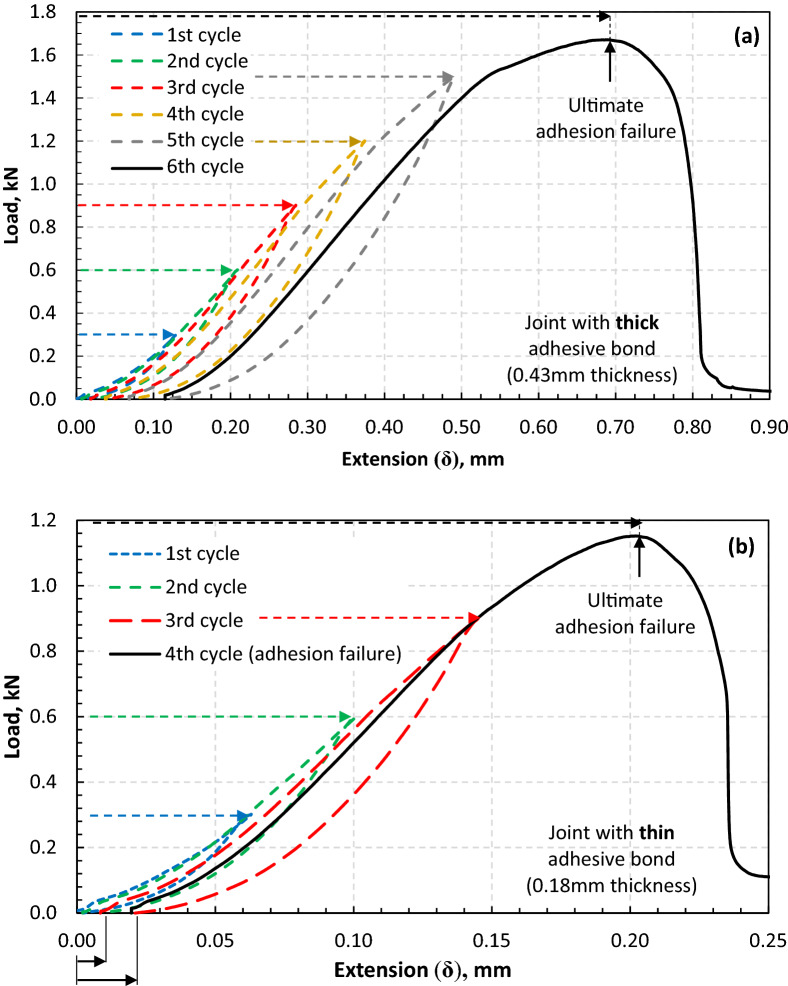
Cyclic tensile data of single-lap bonded joints with CNT modified adhesives for (**a**) thick bond joint after 6 cycles failure (0.43 mm thickness), and (**b**) thin bond joint after 4 cycles failure (0.18 mm thickness); dashed arrows show selected load level for each cycle; solid arrows show residual elongation after unloading for cycle 2 and 3.

The single-lap bonded joints have been subjected to cyclic tensile loads in increments of 300 N to cover a range of linear and non-linear regions (300 N load increments can be identified in the monotonic response data of Fig. 16). Four bonded joint specimens were tested under the proposed cyclic loads with incrementally increasing load of 300 N at each subsequent cycle. One typical load-extension response out of four joint specimens with relatively thin (0.18 mm) and thick (0.43 mm) bonds is shown in Fig. 22, in which the thin bond failed after the 4th cycle, and the thick bond after the 6th cycle. For the first four cycles, the average maximum extensions value per cycle were 0.09 mm, 0.15 mm, 0.20 mm and 0.28 mm, having an almost symmetrical deviation at approx. ± 0.03 mm, ± 0.05 mm, ± 0.07 mm and ± 0.07 mm, respectively. Such symmetrical distribution of the extension data around the average level is due to the fact that the two thick bonds had thickness twice of that in the thin ones, which approximately doubled the bulk adhesive’s elongation data in the thick ones before the ultimate failure. All four specimens exhibited, dominantly, adhesion failure. Residual strains and plastic (inelastic) behaviour is evident in the figure in cycles > 2 where non-zero extensions occurs after unloading. Expectedly, the joint made by the 0.43 mm bond thickness shows higher extension per constant applied load, approximately twice as that in the joint made by the 0.18 mm bond thickness. Note that the volume of adhesive applied to manufacture each type (thin and thick bond) has precisely been controlled as described in “Adhesive preparation and application” section, however after applying higher pressure to make thin bonds, the material has been flown off the edge of the overlap partially on the fillets sides and some having been trimmed off the sides. Therefore the total volume of the adhesive is different in the two types, and the CNT volume fraction has been eventually increased across the thin bond, leading to higher contact points.

**Figure 23:**
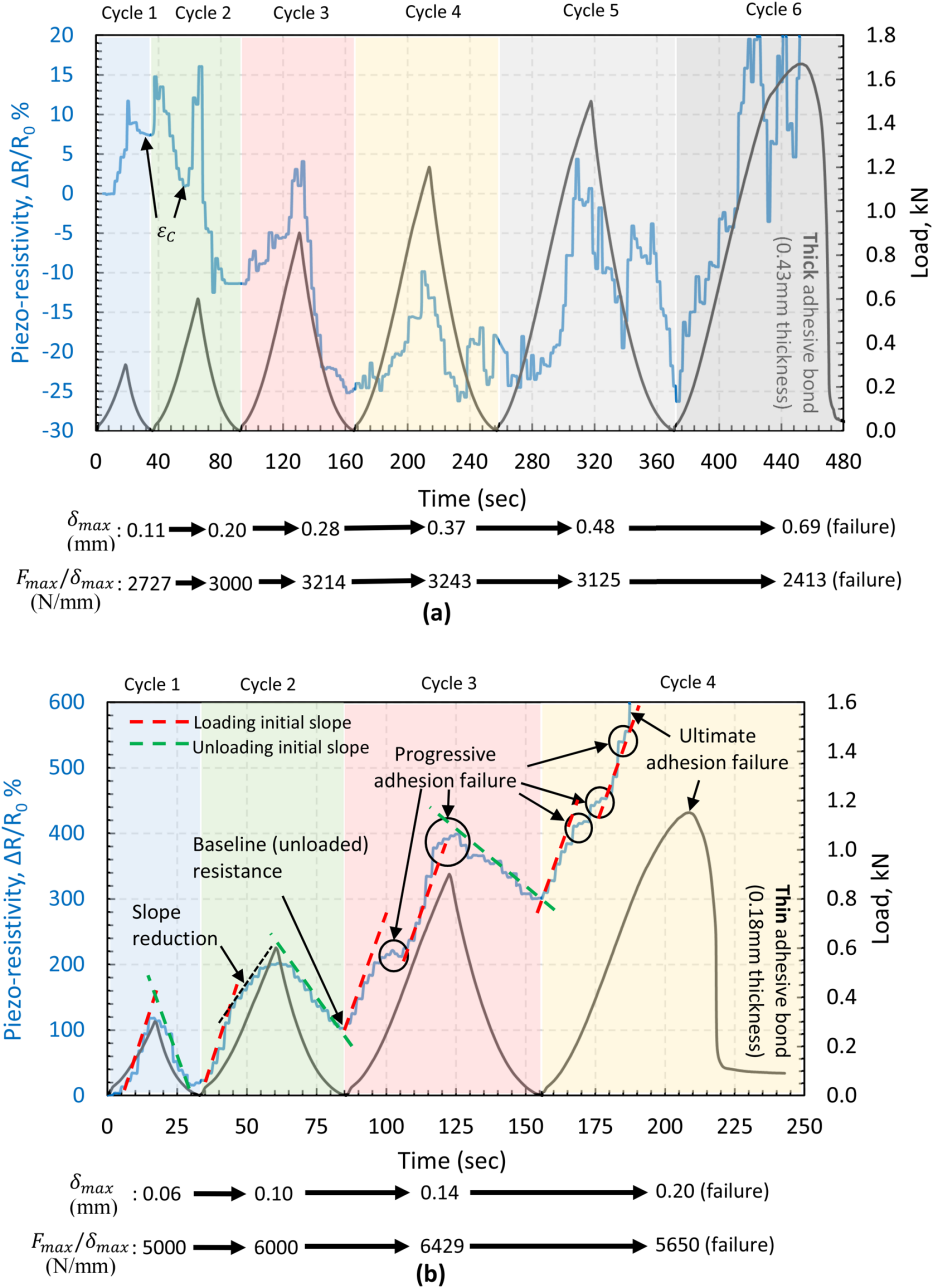
Electrical resistivity response during cyclic tensile loading of single-lap bonded joint specimens in (a) 0.43 mm bond thickness joints, and (b) 0.18 mm bond thickness joints.

The piezo-resistivity data associated with the cycles are plotted in Fig. 23 for the thin (a) and thick (b) bond specimens. As seen, the thick adhesive possesses higher eccentricity which has induced through-the-thickness compressive stresses in the initial cycles (1,2) and consequently the downward-upward phenomenon ($\varepsilon_{C}$) observed formerly in the monolithic response of the thick bonds (Figs. 16, 19). Though the initial resistivity of thick bonds is much higher than that in thin bonds (> 100Ω compared to < 10Ω levels) as a result of less CNT contact points, the difference in $R$ data (${\Delta }R$) is much lower compared to the thin bond which results in a low ${\Delta }R/R_{0}$ levels and in some cycles negative levels. That points out a very poor sensitivity in thick bond joints as opposed to much higher sensitivity and resistivity variations in thin bond joint specimens:

For the thin bonds, less eccentricity is present, and so low compressive levels through the thickness. No downward-upward and $\varepsilon_{C}$ phenomenon is then observed, and a much higher difference in $R$ levels during load evolution has been obtained, and so much higher sensitivity in sensing:

As seen in Fig. 23b, an increasing–decreasing trend is exhibited by the joint in the ${\Delta }R/R_{0}$ correlated with the cyclic loading trend. However, the baseline initial resistance changes during cycles 2–4 since the material undergoes residual strain in those cycles. The ${\Delta }R/R_{0}$ trends are interrupted by damage development (progressive adhesion failure only) in cycle 3 and 4, with direct effect on dramatic increasing of the resistivity. In cycle 4, such dramatic increase is followed by the ultimate failure in adhesion mode. Parameters $\delta_{max}$ and $F_{max} /\delta_{max}$ represents the maximum extension attained at the maximum load in each cycle (denoted by $F_{max}$) and the joint stiffness when $F_{max}$ is reached. The stiffness represents an increasing trend until the ultimate adhesion failure is reached in which the load carrying capacity drops (irrespective of $\delta_{max}$ trend) at which the stiffness level drops simultaneously when the level of ${\Delta }R/R_{0}$ dramatically increases due to full disbond and significant reduction in the conductivity.

Moreover, the ${\Delta }R/R_{0}$ slope in Fig. 23b representative of the sensitivity remains almost constant at the beginning of each cycle irrespective of progressive adhesion failure indicated by creating a resistivity baseline from cycles > 2 (discussed for Fig. 3). Such sensitivity evolution is an indication of a reliable sensing capability via the CNT modified adhesive in thin bonds within the elastic regime (cycle 1) and up to the ultimate failure (cycle 2).

## Conclusions

The ability of the CNT modified adhesive to be used both for in-situ strain measurements and health-monitoring of bonded structures, and specifically for adhesion failure measurement, has been studied in this article. The adhesive’s sensitivity response to varying loads applied to adhesively bonded single-lap joints (all processed to fail in adhesion failure mode only via use of peel ply surface preparation, as observed in Fig. 17) has provided promising indication of adhesion failure in relatively thin bonds (0.18 mm thickness). It has also been shown, that by continuously monitoring the linearity of piezo-resistivity during cyclic loads, the initiation and propagation of damage can be detected prior to the occurrence of the ultimate bond failure. This is an essential quality which could be used for bonded structures with the need for prevention of any unexpected failures, such as in the aviation industry. An interpretation method has been introduced relying upon the resistivity baseline as an outcome of irreversible adhesion crack propagation (mechanism illustrated in Fig. 3 in relation to data presented in Fig. 23a). Moreover, through tensile cyclic tests of adhesive alone, it has been established that the adhesive exhibits a very high sensitivity to strain, more than six times than conventional strain gauges. However, this does not alone lead to a reliable adhesion failure measurement: It has also been observed that strict design considerations are necessary to enable such failure measurement technique since relatively thick bond (0.43 mm thickness) in single-lap joints did not cope with the interpretation method due to the low sensitivity (despite high initial resistivity) and the role of the single-lap eccentricity in introducing through-the-thickness compressive stress at early stages of loading evolution.
