# Author Correction: Carbon nanotube embedded adhesives for real-time monitoring of adhesion failure in high performance adhesively bonded joints

**DOI:** 10.1038/s41598-021-82918-6

**Published:** 2021-02-04

**Authors:** Tadej Bregar, Donglan An, Somayeh Gharavian, Marek Burda, Isidro Durazo-Cardenas, Vijay Kumar Thakur, David Ayre, Marcin Słoma, Mark Hardiman, Conor McCarthy, Hamed Yazdani Nezhad

**Affiliations:** 1 Enhanced Composites and Structures Centre, School of Aerospace, Transport and Manufacturing, Cranfield University, Cranfield, MK43 0AL UK; 2 School of Materials, The University of Manchester, Oxford Road, Manchester, M13 9PL UK; 3 Cametics Cambridge Advanced Metals Limited, Unit 24, South Cambridge Business Park, Babraham Road, Sawston, Cambridge, CB22 3JH UK; 4 Through-Life Engineering Services Institute, School of Aerospace, Transport and Manufacturing, Cranfield University, Cranfield, MK43 0AL UK; 5 Department of Engineering, Science and Technology, SRUC, Edinburgh, DG1 3NE UK; 6 Faculty of Mechatronics, Warsaw University of Technology, 00-661, Warsaw, Poland; 7 School of Engineering, CONFIRM Centre and Bernal Institute, University of Limerick, Limerick, V94 T9PX Ireland; 8 Department of Mechanical Engineering and Aeronautics, City University of London, London, EC1V 0HB UK

Correction to: *Scientific Reports* 10.1038/s41598-020-74076-y, published online 08 October 2020

The Acknowledgements section in this Article is incomplete.

“The authors would like to acknowledge supports from Cametics Ltd., the UK EPSRC funded research (STRAINcomp; Ref. EP/R016828/1). This publication has emanated from research conducted with the financial support of Science Foundation Ireland (SFI) under Grant Number SFI 13/IA/1833 & SFI 16/RC/3918, co-funded by the European Regional Development Fund. The authors are also grateful to Mr. Jim Hurley and Mr. Ben Hopper from Cranfield University for strong and continuous technical supports throughout the research. The underpinning data can be accessed at https://doi.org/10.17862/cranfield.rd.13006631.v1.”

should read:

“The authors would like to acknowledge supports from Cametics Ltd., the UK EPSRC funded research (STRAINcomp; Ref. EP/R016828/1). This publication has emanated from research conducted with the financial support of Science Foundation Ireland (SFI) under Grant Number SFI 13/IA/1833 & SFI 16/RC/3918 and Foundation for Polish Science (FNP) under Grant Number First TEAM/2016–1/7, co-funded by the European Regional Development Fund. The authors are also grateful to Mr. Jim Hurley and Mr. Ben Hopper from Cranfield University for strong and continuous technical supports throughout the research. The underpinning data can be accessed at https://doi.org/10.17862/cranfield.rd.13006631.v1.”

